# Exploring pharmacists' perspectives about substandard and falsified medical products through interviews

**DOI:** 10.1016/j.rcsop.2024.100421

**Published:** 2024-02-14

**Authors:** A. Persson, M. Troein, S. Lundin, P. Midlöv, C. Lenander

**Affiliations:** a Center for Primary Health Care Research, Department of Clinical Sciences Malmö, Lund University, Box 50332, SE-20213 Malmö, Sweden; b Department of Arts and Cultural Sciences, Lund University, LUX, Helgonavägen 3, SE-22100 Lund, Sweden

**Keywords:** Public health, Drug-related side-effects and adverse reactions, Qualitative research, Substandard falsified medical products, Pharmacists, Professional-patient relations

## Abstract

**Background:**

The problem with substandard and falsified (SF) medical products may grow in high-income countries when e-commerce of medicines increases. Unauthorized websites offer medicines of insufficient quality. This underscores the importance of evaluating how the problem with SF medical products can be prevented from escalating. However, little is known about what knowledge and experience professionals working primarily with medicines have about the phenomenon.

**Objective:**

This study was conducted to explore purposively selected pharmacists' experience and knowledge about SF medical products.

**Methods:**

Twelve individual interviews were conducted with purposively selected pharmacists between May 2021 and September 2021. An interview guide was used with specific questions about e-commerce, which focused on exploring pharmacists' experience and knowledge about SF medical products. The interviews lasted, on average, 49 min and were analyzed using inductive qualitative content analysis.

**Results:**

A main theme ‘Pharmacists as guardians of safe medicines’ emerged. This theme consisted of three categories pinpointing ‘risk factors’, ‘protective factors’, and ‘opportunities for improvement’ regarding SF medical products. Findings suggest that pharmacists can play a role in preventing the problem with SF medical products from escalating. Participants emphasized they were in this line of work to help patients and increase patient safety.

**Conclusions:**

Pharmacists have the opportunity to empower the public with knowledge about SF medical products since they discuss medicines with many people every day. Awareness of risk factors for SF medical products enables pharmacists to guide patients to avoid risky purchases from unauthorized websites. To do this, better communication, and cooperation with patients and other healthcare professionals are needed.

## Highlights

• Pharmacists have an important role in raising awareness of SF medicines.
• Pharmacists who are aware of risk situations can identify who needs information.
• Pharmacists, other healthcare professions and patients need to cooperate.

## 1 Introduction

According to the World Health Organization (WHO), substandard and falsified (SF) medical products are one of the urgent health challenges forecast for the next decade.^1^ One of the main access points to SF medical products in high-income countries is via e-commerce.^2^ An evaluation of online websites selling medicines was conducted by the National Association of Boards of Pharmacy, which showed that 95.8% of the 11,688 reviewed websites were unauthorized.^3^ Estimates show that 50% of all medicines sold online from unauthorized websites are SF medical products.^4^

According to the Convert Group, legal e-commerce of medicines is rising, and Sweden is the leading country.^5^ Although Interpol's global ‘Operation Pangea’ indicates a rise in illegal e-commerce of medicines, no corresponding figures for the illegal online trade of medicines are available.^6^ Sweden, a high-income country in the European Union (EU), has set a national goal to be the best in the world at using the opportunities that digitization and eHealth offer by 2025.^7^ For example at present, 99% of all prescriptions in Sweden are electronic and can be filled by all authorized Swedish pharmacies.^8^ Overall, Swedes are used to digitization and eHealth which comes with opportunities but can also increase the risk of SF medical products unless an authorized pharmacy is identified by the patient. Moreover, the EU Falsified Medicines Directive (FMD) was implemented to prevent SF medical products from entering the legal supply chain.^9^ In short, the FMD includes a unique QR code on every package of medicine, which enables traceability from the manufacturer until the medicine is dispensed at the pharmacy.

Although Sweden is the leading country regarding legal e-commerce of over the counter and prescription medicines, the vast majority of medicines are still dispensed in community pharmacies.^10^ The pharmacies have been assigned a threefold social mission by the government, namely to (i) ensure access to medicines, (ii) provide expert and individually tailored information and advice, and (iii) implement and provide information about generic replacements.^10^ Prescription-only medicines are subsidized (with few exceptions) for all Swedish citizens.^11^ This means that Swedes have a maximum level of expenditure for prescription-only medicines during a 12-month period (2400 SEK in 2021; approximately 240 USD). To summarize, two main wholesalers supply Swedish pharmacies and during opening hours at least one pharmacist needs to be attending the pharmacy.^10^

Pharmacists are the experts of medicines in society, working in the entire pharmaceutical chain from research and manufacturing until the medicines are dispensed to the user. At community pharmacies, a pharmacist discusses and dispenses medicine to, on average, 55 patients daily.^10^ Medicine-related issues are the main topic for the profession, and this includes information about why patients buy medicines online.

WHO lists three contributory factors to the emergence of SF medical products, namely constrained access, poor governance, and weak technical capacity.^12^ However, it remains unknown if these three global causes apply in high-income countries such as Sweden. To counteract the problem with SF medical products from escalating in high-income countries, it is important to understand the reasons why people buy medicines outside the legal market.

A search in three leading medical databases (PubMed, CINAHL and Web of Science) was conducted. Cross-sectional surveys with randomly selected pharmacists from high-income countries were found.^13^, ^14^, ^15^, ^16^ However, no studies were found with purposively selected pharmacists that aimed to explore knowledge and experience of SF medical products. This study was conducted to address this evidence gap.

## 2 Methods

### 2.1 Study design

A qualitative design was used where pharmacists participated in individual interviews regarding SF medical products. This manuscript was written in accordance with the COREQ guidelines for reporting qualitative research.^17^

### 2.2 Ethics

An application for ethical review was submitted but was judged not to be required according to the Ethics Review Act by the Swedish Ethical Review Authority (reference number 2019–05011). All participants received written information about the study and were able to ask questions before the interviews. Furthermore, all participants gave oral consent for participation and publication.

### 2.3 Sampling

In a previous digital questionnaire about Swedish community pharmacy employees' knowledge and experience of SF medical products,^15^ 21 pharmacists expressed interest in participating in an interview. The inclusion criteria in the previous study were being aged 18 years or older and employed in a Swedish community pharmacy, working with patients. Of the pharmacists who expressed interest in participating, purposive sampling was applied to ensure diversity based on specific baseline characteristics such as gender, size and location of workplace, and years working in a community pharmacy - see Table 1. Besides the predefined criteria used during the purposive sampling, most of the participants had previous experience in other pharmaceutical areas such as research, pharmaceutical industry, and authority work. One person at a time was selected, contacted, and interviewed. Of the 21 pharmacists who expressed interest, 15 were contacted. Of these, 10 accepted and were interviewed and 5 declined to participate. The remaining 6 pharmacists were never offered to participate since they shared the same predefined features with already interviewed participants. Instead, two additional pharmacists were recruited via snowball sampling, outside of the 21 who initially expressed interest through the previous questionnaire. The reason was to ensure diversity regarding size and location of workplace.

**Table 1:** Baseline characteristics of the participants.

Respondent	Gender	Size^1^ of community pharmacy	Location of community pharmacy	Years working in a community pharmacy
A	Female	Medium	Hospital	24
B	Male	N/A	Ambulatory	10
C	Female	Small	Urban	7
D	Female	Large	Hospital	14
E	Female	Small	Rural	11
F	Male	Medium	Hospital	12
G	Male	Large	Urban	15
H	Male	Large	Urban	15
I	Female	Large	Urban	7
J	Female	Small	Rural	8
K	Male	N/A	Ambulatory	6
L	Male	Medium	Urban	11

N/A not applicable.

1 Small <5 employees, Medium 5 to 9 employees, Large ≥10 employees.

### 2.4 Setting and data collection

Participants chose the time and place for the interviews. Due to geographical distance, most interviews were conducted online via Zoom, with both audio and video enabled. AP, a clinical pharmacist and PhD candidate, with experience in qualitative research gained through a PhD course in qualitative methods, conducted all interviews. The participants knew that AP was not currently working in a community pharmacy. The interviews were conducted using a semi-structured interview guide - see Appendix I. The interview guide was developed to focus on exploring pharmacists' knowledge of and experience with SF medical products. After each interview, the interview guide was evaluated. No major adjustments were made. The interviews were conducted between May 10, 2021, and September 30, 2021. At the beginning of the interviews, each participant was asked to describe their background as a pharmacist as well as a typical day at the community pharmacy, focusing on patient dialogue. The intention behind questions one and two was to break the ice between the interviewer and participant. No compensation for participation was given. All interviews were audio recorded and transcribed verbatim immediately after the interview. The transcribed interviews were anonymized. On average, the interviews lasted 49 min, ranging from 29 to 97 min.

### 2.5 Analysis

Interview transcripts were analyzed continuously using an inductive qualitative content analysis by AP and CL.^18^ CL is a PhD pharmacist and has experience in qualitative research. First, the transcripts were read through multiple times to become familiar with the content. During this preparation phase meaning units were identified by AP and CL separately. The identified meaning units were then discussed until consensus was reached. In the following step, meaning units were condensed and coded by AP. AP and CL discussed the condensed meaning units and codes until consensus was reached. This process was repeated after each interview. Codes from all available interviews were then organized and reorganized after each interview. Sub-categories and categories emerged from the initial codes thus forming a main theme. Interviewing continued until saturation was perceived. To confirm saturation 2 more interviews were conducted.

MT, an MD and professor in family medicine with substantial experience in qualitative research, then evaluated the preliminary analysis. MT received all transcribed interviews, a spreadsheet with meaning units, condensed meaning units, codes, sub-categories, categories, and the main theme. See Table 2 for an example. MT compared and confirmed the analysis with the content of the interviews. Minor changes were made, for example changing the names of sub-categories, categories, and main theme.

**Table 2:** Example of text condensation and coding.

**Main theme**	Pharmacists as guardians of safe medicines		
**↑**			
**Categories**	Risk factors	Protective factors	Opportunities for improvement
**↑**			
**Example of sub-categories**	Limited access to medicines	Pharmacists' control	Communication and cooperation
**↑**			
**Example of codes**	Too expensive	Pharmacists' competence	Building trust
**↑**			
**Example of condensed meaning unit**	Patients who think it is too expensive at the pharmacy say that they will buy online	Worth waiting at the pharmacy	Show interest and develop trust – that will lead to questions
**↑**			
**Example of meaning unit**	Some patients say that they think it [the medicine] is too expensive at the pharmacy. It is just a click away [online]. I don't know how they dare. Some patients are open and talk about it, some do not (C39)	I have heard patients, so many times, say “you will have to wait [at the pharmacy] but it is always worth it” (J239)	If you [the pharmacist] show interest and start a dialogue, then you create trust and then they [the patients] will ask (I120)

## 3 Results

### 3.1 Participants

Six men and six women were interviewed. Participants were, on average, 43 years old, ranging from 30 to 56 years. Nine of the participants had a master's degree and 3 had a bachelor's degree in pharmacy. One person also had a licentiate degree, and another one had a PhD. At the time of the interview, the participants had different responsibilities at the community pharmacies, but all dispensed medicines to patients. The responsibilities were pharmacy manager, compliance with laws and regulations, pharmaceutical knowledge, and stock-keeping.

### 3.2 Main theme

A main theme emerged **‘**Pharmacists as guardians of safe medicines’. All participants stated they were confident they were dispensing medicines of good quality at their community pharmacy. At the same time, they pointed out the importance of being aware of potential risk factors when patients could end up buying medicines from unsafe sources.

### 3.3 Categories

The main theme consisted of three categories, each with sub-categories. The categories with their sub-categories are elaborated below. Codes building up the subcategories are written in bold characters and are, in some cases, illustrated with representative quotes in italics. The quotes are translated from Swedish by the authors.

#### 3.3.1 Risk factors

The first category describes risk factors that may drive patients to make unsafe purchases of medicines. According to participants, this increases the risk of SF medical products.

##### 3.3.1.1 Limited access to medicines

Access can be limited because medicines are regarded as **too expensive** by patients. This often coincided with the fact that some medicines are not subsidized. Another reason can be that a **prescription** is **required** in Sweden but not in other countries. Yet another reason can be that the medicine of choice is **out of stock**. These three codes could cause the patient to look for the medicine on unsafe websites. The participants considered the need for a prescription and/or high price to be more common reasons than the medicine being out of stock. This is reflected in the quote below:

*“Melatonin has raised a lot of questions [at the pharmacy]. One patient asked “Why do I need a prescription? I don't in Great Britain. And why is it so expensive? It is not addictive” B152.*

##### 3.3.1.2 Patients' choices

According to participants, some patients wanted the **freedom to choose** medicine by themselves, without being diagnosed and prescribed a medicine by a physician. Some patients wanted the opportunity to **choose other providers** than community pharmacies. Participants perceived these choices sometimes to be based on a **lack of knowledge** about medicines or **attitudes toward generic replacement of medicines in pharmacies**. Patients' attitudes to generic replacement divided the participants. Half expressed that generic replacement is a risk factor for SF medical products, as shown in the quote below. The other participants claimed that patients currently are used to generic replacement and that it is not a risk factor nowadays. All agreed that discussions about generic replacement with patients consumed valuable time that could be used to provide expert and individually tailored information and advice about medicines.

*“I believe it is fascinating that patients can buy anything [online] but when I suggest another manufacturer of the same medicine at the pharmacy, they think it is fraud” D14.*

The participants identified the greatest risk for unsafe purchases if patients were exposed to limited access (as described in 3.3.1.1 above) and then decided independently to buy the medicine online, without consulting healthcare professionals and pharmacists:

*“I had a dialogue with a patient who had a problem with her thyroid. She had found something from another country, not approved in Sweden. She wanted the physician to apply to the Medical Product Agency to prescribe it [as a special license], but the physician did not think that it was a good idea. So, she bought it from Thailand […] She decided what she wanted and then she felt safe” C200.*

#### 3.3.2 Protective factors

The second category accounts for factors that may protect patients from SF medical products.

##### 3.3.2.1 Authorities' control

The work from authorities, both nationally and within the EU, was believed to counteract SF medical products from entering the legal supply chain. All participants mentioned the EU Falsified Medicines Directive (FMD)^9^ as a protective factor. This, in cooperation with Sweden's unique system, with mainly two **wholesalers of medicines** to the pharmacies and the **national control system,** resulted in participants expressing that medicines of good quality are dispensed at Swedish community pharmacies, i.e., the **pharmacists have trust in the control system**.

*“QR codes have been introduced […] every packaging has one […] when we dispense this package it's deducted from a European database so it can't be sold twice. Then we have a wholesaler oligopoly […] which I think is good regarding SF medical products. In other countries, you have a lot of wholesalers to choose from” F23.*

Participants perceived that protective factors due to the authorities' control took place without the patients' knowledge.

##### 3.3.2.2 Pharmacists' control

**Pharmacists' competence** is an **opportunity for information about medicines**, for example how to use medicines in the optimal way, where to buy medicines and how to store them. Many of the participants described being shown and asked about medicines not bought on the Swedish market. They also emphasized the importance of their competence since:

*“I am in this business to improve safe use of medicines” H145.*

and

*“You need a certain minimum level of competence to maintain patient safety” I146.*

In addition, pharmacies are a place where patients can **return unused medicines**. Some of the unused medicines were clearly purchased from other venues than authorized pharmacies, as evident in the quote below.

*“A patient came with a lot of antibiotics, [which the patient wanted to hand in] many unopened packages […] Czech or German, no labels on and cellophane around. It was a stock delivery […] interestingly almost all from the same batch with a soon-to-come expiry date” B58.*

Participants agreed that the return of unused medicines was important for security reasons and for the sake of the environment. Therefore, when patients returned unused medicines, which were not bought in a Swedish pharmacy, the participants all agreed this was not a situation suited for a dialogue about SF medical products. The reason for this assessment was that patients could be discouraged from returning unused medicines to the pharmacy in the future.

#### 3.3.3 Opportunities for improvement

In this last category, participants proposed interventions that might increase patient safety and decrease the spread of SF medical products.

##### 3.3.3.1 Communication and cooperation

Participants expressed that communication and cooperation with the public and other healthcare professions have the potential to prevent patients from purchasing medicines from unauthorized pharmacies. The main code was about **building trust**, but sometimes **barriers to communication** were expressed**.** Participants mentioned that trust is built by listening to patients and colleagues, not judging, and asking open-ended questions. Trust is also built when pharmacists and physicians mutually reinforce each other's messages to patients, explaining why they choose or do not choose to prescribe or dispense a specific medicine. Especially, it is important to have a communicated plan regarding addictive medicines. This plan should be communicated from physician to patient and address treatment time and expected effects. If trust is not built, there may be barriers to communication and unsafe purchases can be difficult to prevent. A participant gave an example of a patient who was prescribed addictive medicines without a plan for stepping down. When the physician stopped the prescriptions, the patient was not open to communication and the participant had no chance for counseling.

*“In this case, we [pharmacists], as a part of the care team, have failed. They [the patients] did not ask to be addicted. They got a push into it by healthcare” B212.*

##### 3.3.3.2 Prevention

One suggestion to increase awareness about SF medical products was more **public awareness campaigns** in community pharmacies or the media.

##### 3.3.3.3 Framework for the business of pharmacies

Seven of the participants felt they did not have the time to **focus on the pharmacies' main objectives,** i.e., the social mission**.** In particular, it was the individually tailored education to the patient that was not completed. The reason expressed was that such services weren't profitable for the pharmacy. One participant said:

*“Do we [the society] want pharmacies that focus on advising the public about medicines or do we want pharmacies where the employees are forced to sell other products [than medicines to survive economically]? It is not the companies' fault forcing the employees to (do) this as I see it; it is the framework from the government” H143.*

The remaining five participants acknowledged the situation but felt confident in their role as a pharmacist, ensuring they prioritized patients in need of information.

## 4 Discussion

A main theme emerged from the interviews, *‘*Pharmacists as guardians of safe medicines***’***. This theme included three categories: risk factors, protective factors, and opportunities for improvement regarding SF medical products. This study suggests that pharmacists can play a role in preventing the problem with SF medical products from escalating. Participants expressed that they are in this line of work to help patients and increase patient safety. Healthcare professionals can do this in various ways, for example during individual consultation and through cooperation with other people working in healthcare. The awareness of risk factors for purchases from unauthorized websites may prompt pharmacists to pay extra attention to patients at risk.

### 4.1 What is already known on this topic

Risk factors for SF medical products have previously been described.^2^^,^^12^ The risk factors presented by the WHO address the supply side, i.e., where SF medical products are most likely to be found due to constrained access, poor governance, and weak technical capacity. Participants' experience described in the present study touches upon all three causes. Primary motivating factors that could lead patients, i.e., the demand side, to purchase medicines online have been suggested by Mackey & Nayyar.^2^ These were cost, convenience, and greater autonomy. Participants in the present study confirmed that some patients react to the price of medicines, and some want to decide both which medicines to use and where to buy them.

### 4.2 What this study adds

Compared to the WHO's risk factors of poor governance and weak technical capacity, good governance and high technical capacity were described as protective factors by the participants in this study. An example given was the implementation of the EU's FMD which demands both good governance and high technical capacity. This indicates that poor governance and weak technical capacity are not prominent causes of SF medical products in high-income countries such as Sweden.

According to Mendoza, e-commerce with medicines requires high public awareness.^19^ Participants perceived that patients' decisions to buy medicines from other venues than authorized were sometimes based on a lack of knowledge. Sjöstrand et al., argue that to make an autonomous decision information is needed.^20^ According to Orizio, self-diagnosis and self-prescribing are clear and significant patient safety risks.^21^ This study suggests pharmacists should empower their patients with the knowledge needed to prevent online purchases from unauthorized websites. This can be done via individually tailored communication. To prioritize patients in need of information, knowledge about risk factors is necessary. Pharmacists also need to communicate and cooperate with both patients and healthcare professions to find an optimal solution for the patient in question.

It has previously been suggested that pharmacists have an important role to play in counteracting SF medical products.^22^^,^^23^ These studies have focused on the pharmacist’s role in supplying quality medicines. This study adds that pharmacists can play an active role in increasing patients' awareness of SF medical products, i.e., working not only with the supply side but also the demand side. Pharmacists can also cooperate with other healthcare professionals to maintain treatment with high-quality medicines. This is in accordance with the vision of the Pharmaceutical International Federation.^24^

To achieve a reality where pharmacists can empower patients with information regarding safe e-commerce, the general pharmacist needs to gain knowledge identified in this study.

### 4.3 Limitations of this study

The most important limitation of this qualitative study is that results cannot be generalized. The participants were purposively selected to gain a rich understanding of the study topic. Their knowledge and experience regarding SF medical products may not reflect the knowledge and experience of a pharmacist in general. Nevertheless, results can be used to enhance the knowledge of pharmacists in Sweden and other high-income countries.

To establish trustworthiness in this study detailed information is provided about the process. Strengths of this study are the purposive sampling and the triangulation used in the analysis. This increases the credibility of the study. A comprehensive description of the Swedish context is provided to help the reader assess the relevance to their own context.

## 5 Conclusion

In conclusion, this study identifies “Pharmacists as guardians of safe medicines”. Pharmacists have the opportunity to empower the public with knowledge about SF medical products since they discuss medicines with many people every day. Awareness of risk factors for SF medical products enables pharmacists to guide patients to avoid risky purchases from unauthorized websites. Participants wanted to do so by shifting focus from selling goods that are not medicines, toward discussing how to buy and use medicines safely with patients. To do this, better communication, and cooperation with patients and other healthcare professionals are needed. Participants emphasized that they were in this line of work to increase patient safety.

## Funding

This work was supported by the Lund Society of Medicine, Elsa Lundberg and Greta Fleron's fund for studies of adverse drug reactions [grant number 2022/305] and Marcus and Amalia Wallenberg Foundation [grant number 2020/0004]. The funding sources were not involved in any step of the research process.

## CRediT authorship contribution statement

**A. Persson:** Conceptualization, Data curation, Formal analysis, Funding acquisition, Investigation, Methodology, Project administration, Writing – original draft. **M. Troein:** Conceptualization, Formal analysis, Methodology, Supervision, Validation, Writing – review & editing. **S. Lundin:** Conceptualization, Funding acquisition, Methodology, Supervision, Writing – review & editing. **P. Midlöv:** Conceptualization, Methodology, Supervision, Writing – review & editing. **C. Lenander:** Conceptualization, Formal analysis, Methodology, Supervision, Writing – review & editing.

## Declaration of competing interest

The authors declare that they have no known competing financial interests or personal relationships that could have appeared to influence the work reported in this paper.

## Data Availability

The data underlying this article will be shared on reasonable request to the corresponding author.

## Appendix A Supplementary data

Interview guide Image 1
