# Supplementary material for: Exploring pharmacists' perspectives about substandard and falsified medical products through interviews
Source: Explor Res Clin Soc Pharm. 2024 Feb 14;13:100421. doi: 10.1016/j.rcsop.2024.100421 (PMC10885592; doi:10.1016/j.rcsop.2024.100421)
Supplement: Supplementary file 1 — Interview guide [file mmc1.docx]

**Semi-structured guide to interviews**

*Before the interview*

- begin informally and be relaxed
- describe the purpose of the interview and approximately how long it takes
- tell the interviewee that:
- it is ok to cancel the interview at any time without any consequences
- there are no right or wrong answers, I am interested in what you have to say
- encourage the interviewee to elaborate on their answers
- ask about background information, see the last page
- emphasize that everything is confidential
- ask for permission to record
- ask if the interviewee has any questions before the interview starts

*The interview*

- start recording
- begin with the phrase “Let’s begin the interview with XX, it is the DD/YY and we are in PLACE/on ZOOM”

See semi-structured questions on the next page

*After the interview*

- leave your contact information to the interviewee
- thank the interviewee for participating

| **Question:** | **Probing areas:** |
| --- | --- |
| Tell me shortly about your professional background as a pharmacist? | - What do you mean when you say…? - Tell me more about…? - What did you think when…? |
| Describe a typical day at the pharmacy – what do you talk to the patients about? | - What do you mean when you say…? - Tell me more about…? - What did you think when…? |
| You declared your interest in participating via the earlier digital questionnaire. Do you remember your thoughts about SF medical products before that questionnaire?  (Not relevant if recruited via snowball sampling) | - What do you think about SF medical products today? |
| What made you interested in participating? (own experience, other incident) | - How did you get this knowledge? - How do you discuss SF medical products at work? |
| I am very interested in SF medical products… (quiet) | - What do you mean when you say…? - Tell me more about…? - What did you think when…? |
| What are your thoughts about the pharmacies’ threefold social mission*, especially the part about providing expert and individually tailored information and advice? | - What do you mean when you say…? - Tell me more about…? - What did you think when…? |
| What are your thoughts if I argue that the pharmacy system in Sweden is perceived safe by both patients and employees? | - What do you mean when you say…? - Tell me more about…? - What did you think when…? |
| What are your thoughts about buying prescription-only medications online without a prescription? | - What do you mean when you say…? - Tell me more about…? - What did you think when…? |
| How do you think these purchases could be reduced or avoided? | - What do you mean when you say…? - Tell me more about…? - What did you think when…? |
| Anything else you would like to add? | - What do you mean when you say…? - Tell me more about…? - What did you think when…? |
| Do you know anyone else, working in a community pharmacy, who could supply me with valuable information about SF medical products? |  |

* (i) ensure access to medicines (ii) provide expert and individually tailored information and advice (iii) implement and provide information about generic replacement

| Code for interview |  |
| --- | --- |
| Name |  |
| Age |  |
| Gender |  |
| Education |  |
| Country of education |  |
| Position at the pharmacy |  |
| Number of years working at community pharmacies |  |
| Pharmacy location |  |
| Size of pharmacy |  |
| Geographic part of Sweden |  |
| Contact information |  |
